# Supplementary material for: Development of a computer-aided design software for the quantitative evaluation of aesthetic damage
Source: PLoS One. 2019 Dec 18;14(12):e0226322. doi: 10.1371/journal.pone.0226322 (PMC6919621; doi:10.1371/journal.pone.0226322)
Supplement: S1 Code site — 1. (ZIP) [file pone.0226322.s001.zip › [EESC_jr][Projeto]Relatorio_07_21/reports/templates_zip/gentelella-master/vendors/jQuery-Smart-Wizard/smartwizard2-validation.htm]

Smart Wizard 2 - Basic Example - a javascript jQuery wizard control plugin

# Smart Wizard 3

## a javascript jQuery wizard control plugin

Download
Documentation
Github

### Examples:

Basic Example
Vertical Style
Multiple Wizards
Ajax Contents
Step Validation

## Example: Step validation

Please
download
and look in the
`more_examples`
folder for an example of validating the steps of the wizard.
